# Deconstructing Hyperlactatemia in Sepsis Using Central Venous Oxygen Saturation and Base Deficit

**DOI:** 10.1164/rccm.201904-0899ED

**Published:** 2019-09-01

**Authors:** Matthew W. Semler, Mervyn Singer

**Affiliations:** ^1^ Division of Allergy, Pulmonary, and Critical Care Medicine Vanderbilt University Medical Center Nashville, Tennessee and; ^2^ Bloomsbury Institute of Intensive Care Medicine University College London London, UK

For over half a century, clinicians and researchers have endeavored to understand the relationship between oxygen delivery and lactic acidosis (1, 2) (or, as discussed below, perhaps more readily considered as “hyperlactatemia with or without acidemia”). In health, pyruvate (the generally acknowledged end product of glycolysis) is metabolized by mitochondria to acetyl coenzyme A to feed the tricarboxylic acid cycle. Excess pyruvate is reduced by lactate dehydrogenase to l-lactate. Notably, this reduction consumes a proton: pyruvate + NADH + H^+^ ↔ lactate + NAD^+^. Lactate is subsequently oxidized back to pyruvate, either locally or after transfer to organs that use lactate as a fuel source (e.g., liver, kidney, and brain) or that convert it back to glucose (the Cori cycle in the liver). In concert, these processes maintain normal blood lactate levels.

During sepsis, lactate levels frequently rise. Indeed, hyperlactatemia (a measurable surrogate for cellular/metabolic perturbations) is closely associated with sepsis prognosis and is now one of the criteria for septic shock (3). However, it remains challenging to determine clinically when a persistently elevated serum lactate level indicates ongoing inadequacy of oxygen delivery, or when the problem lies elsewhere. The brainstem response to give yet more fluid is often inappropriate and potentially injurious.

Hyperlactatemia during sepsis may result from anaerobic glycolysis. When whole-body oxygen delivery fails to meet cellular demands, tissues transition from predominant mitochondrial aerobic respiration to less efficient ATP generation by glycolysis. This is most commonly observed at the time of initial patient presentation, and in many cases can be resolved by administration of intravenous fluids with or without vasoactive agents. However, other factors may also increase serum lactate levels in sepsis, including β_2_-receptor stimulation from endogenous/exogenous catecholamines, impaired tissue oxygen extraction (mitochondrial dysfunction with or without microcirculatory dysfunction), liver dysfunction, and thiamine deficiency.

To aid the clinician in his/her decision-making, Gattinoni and colleagues (pp. 582–589) in this issue of the *Journal* propose a conceptual model relating oxygen delivery and utilization, serum lactate concentration, and acidemia (4). They analyzed data from 1,741 ICU patients who were enrolled in the ALBIOS (Albumin Italian Outcome Sepsis) trial, using serum lactate, central venous oxygen saturation (Scv_O_2__), and blood gas measurements taken at study enrollment (5). Fundamentally, their proposed model frames two clinical questions:

1. Is an elevated lactate level due to inadequate oxygen delivery and therefore potentially responsive to interventions that increase oxygen delivery?
2. How does an elevated serum lactate level affect arterial pH and base excess?

## Hyperlactatemia and Scv_O_2__

High values of Scv_O_2__ suggest systemic oxygen delivery in excess of oxygen demands, impaired cellular (mitochondrial) oxygen use, and/or microcirculatory shunting. Low Scv_O_2__ values imply inadequate oxygen delivery that fails to meet metabolic demands. Gattinoni and colleagues propose the use of Scv_O_2__ to personalize sepsis management, reserving interventions to increase oxygen delivery to only those patients with low Scv_O_2__ values. Of note, only 35% of patients in the ALBIOS trial had Scv_O_2__ values <70%. Other recent sepsis trials reported similar Scv_O_2__ values after initial resuscitation (6).

This proposal is not inherently novel. Both the concept of early goal-directed therapy (EGDT) (7) and the Surviving Sepsis Campaign recommendations (8) suggest that a low Scv_O_2__ should trigger interventions (e.g., fluid, inotropes, and blood) to increase oxygen delivery. This concept has a strong physiologic rationale, but the devil is in the details.

First, the patients in the ALBIOS study and the three recent EGDT trials (6) were all enrolled *after* initial resuscitation. On first presentation, many patients will have impaired oxygen delivery and thus lower Scv_O_2__ values, and a higher likelihood of responding positively to empiric fluid administration. An important caveat is that a low Scv_O_2__ in sepsis does not automatically equate to hypovolemia. Cardiomyopathy can also contribute, and may be worsened by excessive fluid administration.

Second, many patients with sepsis-associated hyperlactatemia have Scv_O_2__ values that fall within an indeterminate range, and even patients with an elevated Scv_O_2__ may respond physiologically to fluid administration (9). Moreover, Scv_O_2__ is a “global” (or rather an “upper-body”) measure of the oxygen supply/demand balance, and may miss imbalances in specific tissue beds (10).

Finally, the history of sepsis research is paved with physiologically rational interventions that nonetheless failed to improve patient outcomes (11). The recent EGDT trials showed no benefit in targeting Scv_O_2__ even among a subset of patients with baseline values <70% (6). Interventions to increase oxygen delivery may have unintended consequences outside the mechanistic pathway assessed by Scv_O_2__ measurement (12, 13). Therefore, an Scv_O_2__-based strategy to personalize interventions for patients with sepsis-associated hyperlactatemia requires careful evaluation in clinical trials before any recommendation regarding standard-of-care implementation in clinical practice can be made.

## Hyperlactatemia and Arterial pH

According to the “strong ion” theory, lactate is a strong anion and thus should be completely dissociated from hydrogen in plasma, generating an acidosis. However, some patients with sepsis and hyperlactatemia have a concurrently decreased pH (acidemia), whereas others maintain a normal pH. This suggests mechanisms that enable relatively rapid respiratory or metabolic compensation. Gattinoni and colleagues found that the ability to maintain a normal pH despite elevated lactate levels was more closely correlated with renal function than with respiratory compensation. They propose that an indirect measure of the accumulation of renally excreted fixed acids in plasma—the “alactic base excess”—could be used to assess the kidneys’ ability to compensate for acid-base disturbances.

The standard base excess, defined as the amount of strong acid that must be added to each liter of oxygenated blood to return the pH to 7.40 at a Pa_CO_2__ of 40 mm Hg, quantifies the degree of metabolic acidosis or alkalosis independently of respiratory compensation. Contributors to base excess include lactate, strong ions such as sodium and chloride, albumin, and ions that accumulate in renal failure, such as phosphate and sulfate (14). By adding lactate to the standard base excess, the authors arrive at the alactic base excess, which they assert quantifies “the role of renal function in the acid*–*base balance in sepsis.”

This suggestion is certainly interesting but requires further thought and investigation. Renal compensation for acid–base disturbances has traditionally been considered to be slower than respiratory compensation. Detailed data on urine output, stage of acute kidney injury (15), minute ventilation, and other physiologic measures would be required before the relative causal effects of kidney injury in compensating for acidosis could be fully understood. The alactic base excess is not necessarily an explicit measure of renal function. For example, administration of 0.9% sodium chloride decreases the base excess, even in the presence of stable renal function and lactate concentrations (16). The impact of concurrent liver dysfunction requires consideration, and only a few such cases were included in the ALBIOS database. Nonetheless, the concept of alactic base excess and the role of renal function in modifying acidemia warrant evaluation in future physiologic studies.

In summary, Gattinoni and colleagues are to be congratulated for advancing an ambitious conceptual model relating oxygen delivery, lactate generation, renal function, and acidemia in sepsis. We are eager to see future research to confirm and refine this model, and move us closer to the authors’ vision of a more personalized approach to early hemodynamic management for sepsis.

## Supplementary Material

Supplements

Author disclosures
