# Supplementary material for: Deconstructing Hyperlactatemia in Sepsis Using Central Venous Oxygen Saturation and Base Deficit
Source: Am J Respir Crit Care Med. 2019 Sep 1;200(5):526–7. doi: 10.1164/rccm.201904-0899ED (PMC6727157; doi:10.1164/rccm.201904-0899ED)
Supplement: Supplements [file rccm.201904-0899ED.html]

Deconstructing Hyperlactatemia in Sepsis Using Central Venous Oxygen Saturation and Base Deficit | American Journal of Respiratory and Critical Care Medicine

- disclosures.pdf (68 KB)
